# Supplementary material for: Targeting Homologous Recombination Deficiency in Ovarian Cancer with PARP Inhibitors: Synthetic Lethal Strategies That Impact Overall Survival
Source: Cancers (Basel). 2022 Sep 23;14(19):4621. doi: 10.3390/cancers14194621 (PMC9563432; doi:10.3390/cancers14194621)
Supplement: Supplementary file 1 [file cancers-14-04621-s001.zip › cancers-1832471-supplementary.pdf]

Supplementary Table S1. Clinical trials of PARP inhibitors (PARPis) as a monotherapy in ovarian cancer

Trial Name; identifier	Drug Name	Phase; Number of Patients	Time	Country/ Countries	Biomarker(s) for Eligibility	Design	Outcomes
Study 19; NCT00753545 [118,199]	Olaparib	II; N=265	2008-2015	17 countries	N/A	Platinum-sensitive, recurrent HGSOC, received at least 2 platinum-based regimens	OS: 29·8 months [95% CI 26·9-35·7] for olaparib vs 27·8 months [24·9-33·7] for placebo OS: BRCAm 34·9 months [95% CI 29·2-54·6] olaparib vs 30·2 months placebo [23·1-40·7]). OS: BRCAwt 24·5 months [19·8-35·0] olaparib vs 26·6 months [23·1-32·5] placebo). PFS: 8.4 months olaparib vs 4.8 months placebo. [HR] 0.35 (95% CI, 0.25 to 0.49).
Study 42; NCT01078662 ^ [101,199]	Olaparib	II; N=298	2010-current	6 countries	gBRCAm	gBRCAm advanced platinum resistant OC having been treated with ≥3 prior lines of chemotherapy	ORR 31.1% (60 of 193; 95% CI, 24.6 to 38.1)
SOLO-1; NCT01844986 [89,93]	Olaparib	III; N = 391	2013-current	15 countries	BRCAm	BRCAm OC following first line platinum-based chemotherapy	PFS, 56·0 months with olaparib versus 13·8 months with placebo [HR] 0·33 (95% CI 0·25–0·43)
SOLO2/ENGOT-Ov21; NCT01874353 [89,94]	Olaparib	III; N=295	2013-current	16 countries	BRCAm	BRCAm recurrent platinum-sensitive HGSOC or high-grade endometrioid;	OS: 51·7 months (95% CI 41·5-59·1) with olaparib and 38·8 months (31·4-48·6) with placebo [HR] 0·74 (95% CI 0·54-1·00; p=0·054)

SOLO-3; NCT02282020 [200, 201]	Olaparib	III; N=266	2015-current	USA	gBRCAm	gBRCAm platinum-sensitive relapsed OC	ORR: olaparib 72.2% vs. chemotherapy 51.4%, odds ratio [OR], 2.53 (95% CI, 1.40 to 4.58; P = .002).
ORZORA; NCT02476968 [202]	Olaparib	IV; N=181	2015–2021	8 countries	gBRCAm or sBRCAm or mutation in HRR pathway (ATM, BARD1, BRIP1, CDK12, CHEK1, CHEK2, FANCL, PALB2, PPP2R2A, RAD51B, RAD51C, RAD51D, and RAD54L)	Platinum sensitive relapsed BRCAm or HRD OC with complete or partial response following platinum-based chemotherapy	PFS 18.0 months, BRCAm; 16.4 months, non-BRCA HRD); OS 42.6 months in BRCAm and 39.3 months in non-BRCA HRD
CLIO/BGOG-ov10; NCT02822157 [203]	Olaparib	II; N=160	2016-current	Belgium	N/A	Recurrent OC treated with at least one previous line of chemotherapy	ORR: in heavily pretreated PROC (>4 prior lines) was 22.9% (8/35) ORR: 35.7% (5/14) and 13.2% (7/53) in BRCAm and BRCAwt, respectively
OPINION; NCT03402841 [204]	Olaparib	IIIb; N=279	2018-current	17 countries	sBRCAm	sBRCAm OC with platinum-sensitive relapsed ovarian cancer (PSROC) and had received ≥2 previous lines of platinum-based	Median PFS was 9.2 months, (95% CI, 7.6-10.9). PFS was 16.4, 11.1, 9.7, and 7.3 months in sBRCAm, HRD including sBRCAm, HRD excluding sBRCAm, and HRR proficient patients, respectively
OVAL-1; NCT04532645 [201]	Olaparib	N=350	2020-current	France, Italy, UK	BRCAm	BRCAm advanced (FIGO stage III-IV) ovarian cancer patients	

						who received first dose maintenance olaparib	
ARIEL2; NCT01891344 [22]	Rucaparib	II; (Part I N=204 and Part II N=287)	2013–2019	7 countries	BRCAm; tumor genomic LOH (high or low)	Relapsed OC: BRCAm, BRCAwt and LOH high (LOH high group), or BRCAwt and LOH low (LOH low group).	Part I: median PFS: 12.8 months, (9.0–14.7) in BRCAm vs. 5.7 (5.3–7.6) in LOH high group vs 5.2 (3.6–5.5) in LOH low group. PFS is significantly longer in BRCAm [HR] 0.27, (95% CI, 0.16–0.44, P < 0.0001) and LOH high [HR] 0.62, (95% CI 0.42–0.9, P = 0.011). compared to LOH low group. Part II: confirmed ORR: 31% (21.3–42), 6.8% (2.3– 15.3) and 5.6% (2.1– 11.8), respectively. Median PFS: 7.8 months (7.3–9.2) vs 4.3 months (3.5–5.7) vs 4.0 months (3.5–5.3), P < 0.001)
ARIEL3; NCT01968213 [104]	Rucaparib	III; N=564	2014-2016	11 countries	N/A	Relapsed platinum-sensitive, OC	PFS in BRCAm carcinoma was 16·6 months (95% CI 13·4–22·9; 130 [35%] patients) in the rucaparib group versus 5·4 months (3·4–6·7; 66 [35%] patients) in the placebo group [HR] 0·23 [95% CI 0·16–0·34]; p<0·0001). PFS: in patients with a HRD carcinoma (236 [63%] vs 118 [62%]) was 13·6 months (10·9–16·2) versus 5·4 months (5·1–5·6; [HR] 0·32 [95% CI 0·24–0·42]; p<0·0001).

ARIEL4; NCT02855944 [205]	Rucaparib	III; N=349	2017-current	USA	N/A	Relapsed OC. Received ≥ 2 prior chemotherapy regimens and have relapsed or progressive disease	PFS: 7·4 months (95% CI 7·3–9·1) in the rucaparib group versus 5·7 months (5·5–7·3) in the chemotherapy group [HR] 0·64 (95% CI 0·49–0·84]; p=0·0010).
ATHEN; NCT03522246 [206,207]	Rucaparib	III; N=538	2018-current	24 countries	N/A	First-line; Newly diagnosed advanced OC	PFS: 28.7 months (95% CI) with rucaparib versus 11.3 months with placebo in the HRD population (log-rank P = .0004; [HR], 0.47; 95% CI, 0.31 to 0.72)
MAMOC; NCT04227522 [208]	Rucaparib	III; N=190	2020-current	Germany	BRCAm	Maintenance after Bevacizumab maintenance following carboplatin based first line chemotherapy, BRCAm patients with histologically confirmed, advanced OC in first line therapy.	
NOVA; NCT01847274 [103]	Niraparib	III; N=553	2013-current	GSK, US	N/A	Maintenance in platinum sensitive OC patients who have either gBRCAm or a tumor with HGS histology and who have responded to their most recent chemotherapy containing a platinum agent.	PFS: gBRCAm 21.0 months with niraparib vs 5.5 months with placebo<br>PFS: BRCAwt 9.3 months with niraparib vs 3.9 months with placebo [HR] (95% CI) gBRCAm 0.3 (0.2–0.4); BRCAwt 0.5 (0.3–0.6)
QUARA; NCT02354586 [105]	Niraparib	II; N=463	2015-2021	USA/ Canada	BRCAm or HRD positive	HRD platinum-sensitive OC tumors which received three to five	OS: 12.2 months<br>ORR: 24%, 95% CI 16–34%

						previous chemotherapy regimens, and PARPi naïve.	
PRIMA/ENGOT-OV26/GOG-3012 NCT02655016 [97]	Niraparib	III; N=733	2016-current	20 countries	N/A	Maintenance treatment in advanced OC following response on front-line platinum-based chemotherapy	PFS: All 13.8 months niraparib vs 8.2 months placebo PFS: HRD 21.9 months niraparib vs 10.4 months placebo. [HR] (95% CI) All 0.6 (0.5–0.8) HRD 0.4 (0.3–0.6)
NIRVANA-R; NCT04734665 [209]	Niraparib	II; N=44	2021-current	Korea	N/A	PFS (6 months PFS rate) with PS recurrent OC previously treated with a PARPi.	
NCT03509636 [70,114]	Fuzuloparib	I; N=113	2018-2020	China	BRCAm	Platinum-sensitive recurrent OC (disease progression or relapse ≥6 months after the last dose of platinum-based therapy) and BRCAm	ORR: The IRC- and investigator-assessed 69.9% (95% CI, 60.6-78.2) and 70.8% (95% CI, 61.5-79.0), respectively. PFS: The IRC- and investigator-assessed 12.0 months (95% CI, 9.3–13.9) with Fuzuloparib and 10.3 months (95% CI, 9.2–12.0). The 12-month survival rate was 93.7% (95% CI, 87.2–96.9).
NCT03863860 [70]	Fuzuloparib	III; N=252	2019-current	China	N/A	platinum-sensitive recurrent OC after at least two previous lines of platinum-based chemotherapy and achieved either complete or partial response to their most recent regimen	PFS: As of July 1, 2020, the median PFS was significantly improved with Fuzuloparib treatment [HR], 0.25; (95% CI, 0.17 to 0.36; one-sided P < .0001) compared with placebo. Trend of benefit in patients with gBRCAm [HR], 0.14; (95% CI, 0.07 to 0.28) compared to

							BRCAwt [HR], 0.46; (95% CI, 0.29 to 0.74).
NCT03519230 [69]	Pamiparib	III; N=216	2018-current	China	N/A	Platinum-sensitive recurrent OC	
NCT03333915 [69]	Pamiparib	I/II; N=113	2019-current	China	BRCAm	Platinum sensitive or resistance recurrent OC with gBRCAm and > 2 lines therapy	ORR: 31.6%\ OR: 11.1 months PFS: 6.2 months PS OC; ORR: 64.6% PFS: 15.2 months PR OC; ORR: 31.6%, PFS: 6.2 months
NCT00892736 ^ [210]	Veliparib	I; N=98	2009-2017	USA	N/A	Recurrent platinum-refractory OC that progressed following standard therapy or had no standard treatment options	ORR: 23% (95% CI 13–35%) in BRCAm and 8% (95% CI 1–26%) in BRCAwt. CBR: 16% (95% CI 4–36%), reflecting prolonged stable disease in some patients.
NCT01339650 ^ [211]	ABT-767	I; N=93	2011-2017	Netherlands	BRCAm	Recurrent BRCAm advanced solid tumors and in OC	PRS: 6.7 months in HRD versus 1.8 months in HRR proficient patients with OC.
NCT03878849 ^ [212]	E7449 (2X-121)	II; N=60	2019-current	USA/UK	N/A	Advanced OC	

Supplementary Table S2. Clinical trials of PARP inhibitors (PARPis) in combination therapy for ovarian cancer

Trial Name; identifier	Drug Name	Phase; Number of Patients	Time	Country/ Countries	Biomarker(s) for Eligibility	Design	Outcomes
PARPi + chemotherapy							
MITO25; NCT03462212A [213]	Rucaparib + carboplatin/paclitaxel	II; N=244	2018-current	Italy	N/A	Advanced OC	
VELIA; NCT02470585 [214]	Veliparib + Carboplatin/Paclitaxel	III; N=1140	2015-current	USA	N/A	Newly diagnosed stage III or IV OC	PFS: 34.7 (veliparib) vs. 22 (control) in BRCAm-cohort, [HR]0.44, (95% CI: 0.28–0.68). PFS: 31.9 vs. 20.5 in the HRD cohort, [HR] 0.57, (95% CI: 0.43–0.76).
PARPi + DNA Damage Repair inhibitor							
OLAPCO; NCT02576444 ^ [215]	Olaparib + AZD6738 (Ceralasertib; ATR inhibitor)	II; N=24	2015-current	USA	BRCAm or mutations such as ATM, CHK2, MRN (MRE11/NBS1/RAD 50), CDKN2A/B and APOBEC; IDH1/IDH2 mutations; TP53 and/or KRAS mutations; PTEN, PIK3CA, AKT, or ARID1A mutations or other molecular aberrations leading to dysregulation of	Relapsed HGSOC resistant to platinum and PARP inhibitors (1-3 prior agents) harboring HRR mutations and BRCAm.	Of 7 HSGOC patients; 1 achieved partial response, 3 had regression < 30% (1 ongoing at 1 year) and 3 patients had progressive disease

					the PI3K/AKT pathway		
NCT02898207 ^ [88,216]	Olaparib + AT13387 (Onalespib; Hsp90 inhibitor)	I; N=28	2017-current	USA	N/A	Treatment of advanced solid tumors with expansion in patients with recurrent OC	
EFFORT; NCT03579316 [88,217]	Olaparib + AZD1775 (Adavosertib; WEE1 inhibitor)	II; N=80	2018-current	USA	N/A	Recruiting; Recurrent OC	Adavosertib: ORR = 23%; PFS = 5.5 months Adavosertib + Olaparib: ORR = 29%, PFS = 6.8 months
ATARI; NCT04065269 [218]	Olaparib + AZD6738 (ATR inhibitor)	II; N=40	2019-current	UK	ARID1A-deficient ('loss') and "no loss."	Recruiting; Gynecological cancers with ARID1A loss or no loss	
CAPRI; NCT03462342 [219]	Olaparib + AZD6738 (Ceralasertib; ATR inhibitor)	II; N=86	2022-current	USA	N/A	Recruiting; recurrent platinum-sensitive or platinum-resistant OC	PFS 4.2 months overall (90% CI: 3.5-8.2) and 8.2 months for patients with BRCAm
NCT04267939 ^ [218]	Niraparib + BAY1895344 (ATR inhibitor)	I/Ib; N=56	2020-current	USA	N/A	Recruiting; Advanced OC	
NCT04149145 [220]	Niraparib + M4344 (ATR inhibitor)	I; N=40	not yet recruiting	USA	N/A	Recurrent OC that has progressed while on a PARP	
PARPi + PI3K / AKT pathway inhibitors							
NCT01623349 ^ [88,221]	Olaparib + BKM120 (pan-PI3K inhibitor) or BYL719 (selective PI3Kα inhibitor)	I; N=118	2012-2020	USA	N/A	Recurrent HGSOC	Of the 28 patients with OC, ten (36%) achieved a partial response and 14 (50%) had stable disease

NCT02208375 [222]	Olaparib + AZD5363 (Capivasertib; AKT inhibitor)	I/II; N=159	2014-current	USA	BRCAm	BRCAm recurrent OC	ORR = 19%; PFS = 14 months; CBR = 34%
NCT04586335 ^ [88]	Olaparib + CYH33 (PI3Kα inhibitor)	I; N=350	2020-current	USA and Australia	N/A	Recruiting; recurrent platinum resistant or refractory HGSOC	
NCT03586661 [88,222]	Niraparib + Copanlisib (PI3K inhibitor)	Ib; N=44	2019-current	USA	BRCAm	Recruiting; recurrent HGSOC, or deleterious BRCAm recurrent OC	
PARPi + Anti-angiogenic							
NCT01116648 ^ [223]	Olaparib + AZD2171 (Cediranib; VEGF inhibitor)	II; N=155	2010-2018	USA	BRCAm	Recurrent advanced OC	PFS 17.7 months for women with cediranib plus olaparib combination treatment compared with 9.0 months for those with olaparib monotherapy. Significant improvement in PFS gBRCAwt women receiving cediranib/olaparib (16.5 vs. 5.7 mos, p = 0.008) PFS: gBRCAm patients (19.4 vs. 16.5 mos, p = 0.16). ORR: in the 18 OC patients 44%, with CBR (ORR plus SD >24 weeks) of 61%.
PAOLA-1; NCT02477644 [224]	Olaparib + Bevacizumab (VEGF-A inhibitor)	III; (N=806)	2015-2019	11 countries	N/A	First line maintenance; advanced OC treated standard first-line treatment	PFS: 22.1 months vs 16.6 months; [HR], 0.59: (95% CI 0.49 to 0.72); P < 0.001; PFS: 37.2 months vs. 17.7 months; [HR] , 0.33; (95% CI 0.25 to 0.45) in patients with HRD including BRCAm;

							PFS: 28.1 mo vs. 16.6 mo; [HR], 0.43; (95% CI 0.28 to 0.66) in patients with HRD without BRCAm
CONCERTO; NCT02889900 [225]	Olaparib + AZD2171 (Cediranib; VEGF inhibitor	II; N=62	2017-2021	USA	No BRCAm	Non-BRCAm recurrent platinum resistant OC	ORR = 15.3%; median PFS = 5.1 months; median duration of response = 8.3 months; median OS = 13.2 months.
NCT04229615 [69]	Fuzuloparib + Apatinib (VEGFR2 inhibitor)	III; N=690	2020-current	China	N/A	Recruiting, maintenance therapy in advanced OC following response on first-line platinum-based chemotherapy	
NCT04517357 [69]	Fuzuloparib + Apatinib (VEGFR2 inhibitor)	II; N=142	2020-current	China	N/A	Recruiting; Advanced OC following 2 or more platinum-containing regimens	
PARPi + Histone Deacetylase inhibitor							
NCT04703920 ^ [226]	Talazoparib + Belinostat (HDAC)	I; N=25	2021-current	USA	N/A	Recruiting; metastatic ovarian cancer	
PARPi + Immunotherapy inhibitor							
NCT02734004 ^ [227]	Olaparib + MED14736 (PD-L1 inhibitor)	I/II; N=264	2016-2021	7 countries	N/A	Advanced OC	
NCT02953457 [88]	Olaparib + Tremelimumab (CTLA-4 inhibitor) + Duralumab (PD-L1 inhibitor)	II; N=40	2017-2021	USA	gBRCAm; sBRCAm; HRD LOH high	Recurrent or refractory OC with BRCAm	
NCT04034927 [88]	Olaparib + Tremelimumab (CTLA-4 inhibitor)	II; N=175	2019-current	USA	N/A	Recurrent OC	

NCT02571725 [88,228]	Olaparib + Tremelimumab (CTLA-4 inhibitor)	I/II; N=50	2022-current	USA	gBRCAm	gBRCAm OC	
ATHEN; NCT03522246 [206]	Rucaparib+Nivolumab (PD-1 inhibitor)	III; N= 538	2018-current	24 countries	N/A	First-line; Newly diagnosed advanced OC	
TOPACIO/KEYNOTE-162; NCT02657889 ^ [194,195]	Niraparib + Pembrolizumab (PD-1 inhibitor)	I/II; N=122	2016-2021	USA	N/A	Completed; platinum resistant OC having experienced a response lasting at least 6 months to first-line platinum-based therapy	ORR of 18% [5% complete responses and 13% partial responses} CBR of 65%
MOONSTONE; NCT03955471 [88,229]	Niraparib + TSR-042 (Dostarlimab; PD-1 inhibitor)	II; N=41	2019-current	USA	N/A	Recurrent OC received 1–3 lines of prior therapy (one of which included bevacizumab), and have disease progression <6 months from the last administered platinum-based chemotherapy	
ROCSAN; NCT03651206 [230]	Niraparib + TSR-042 (Dostarlimab; PD-1 inhibitor)	II/III; N=196	2020-current	France	N/A	Recruiting; recurrent or progressing OC after at least a first line of platinum-based chemotherapy	
PARPi + Multiple Combinations							
NCT02484404 ^ [231,232]	Olaparib + Durvalumab (PD-L1 inhibitor) ± Cediranib (VEGF inhibitor)	I/II; N=384	2015-current	USA	N/A	Recruiting; advanced OC	
MEDIOLA; NCT02734004 ^ [227]	Olaparib + MEDI4736 (PD-L1 inhibitor) + Bevacizumab (VEGF-A inhibitor)	I/II; N=264	2016-2021	7 countries	N/A	Advanced OC	
OPAL; NCT03574779 [88]	Niraparib + Dostarlimab (PD-1 inhibitor) +	II; N=125	2018-current	USA	N/A	Active, recruiting; Recurrent and newly diagnosed OC	

	Bevacizumab (VEGF-A inhibitor)					
NCT05065021 [88]	Niraparib + Dostarlimab (PD-1 inhibitor) + Bevacizumab (VEGF-A inhibitor)	II; N=40	2022-current	Canada	N/A	Not yet recruiting; OC patients who previously received PARPi

^ Not only ovarian cancer; Abbreviations: BRCAm, BRCA mutant; BRCAwt, BRCA wild-type; CBR, clinical benefit rate; CTLA-4, cytotoxic T-lymphocyte-associated protein 4; gBRCAm, germline BRCA mutant; HDAC, histone deacetylase; HGSOC, high-grade serous ovarian cancer; HR, Hazard Ratio; HRR, Homologous recombination repair; HRD, Homologous recombination deficient; Hsp90, heat shock protein 90; IRC, independent review committee; LOH, loss of heterozygosity; OC, ovarian cancer; ORR, objective response rate; OS, overall survival; PARPi, PARP inhibitor; PD-1, programmed cell death protein 1; PD-L1, programmed death-ligand 1; PFS, progression free survival; PS, platinum sensitive; PR - platinum resistant; PROC, platinum resistant ovarian cancer; sBRCAm, somatic BRCA mutant; VEGF-A, vascular endothelial growth factor A; VEGFR2, vascular endothelial growth factor receptor 2.
